# Kaposiform lymphangiomatosis—the effects of long-term treatment with sirolimus: case series study and review of the literature

**DOI:** 10.3389/fmed.2026.1705297

**Published:** 2026-04-23

**Authors:** Magdalena Bilny-Paluch, Piotr Piekarczyk, Katarzyna Błasińska, Małgorzata Szołkowska, Mateusz Polaczek, Elżbieta Radzikowska

**Affiliations:** 1 The Third Department of Lung Diseases and Oncology, National Tuberculosis and Lung Diseases Institute, Warsaw, Poland; 2 Department of Radiology, National Tuberculosis and Lung Diseases Institute, Warsaw, Poland; 3 Department of Pathology, National Tuberculosis and Lung Diseases Institute, Warsaw, Poland

**Keywords:** complex lymphatic anomalies, kaposiform lymphangiomatosis, lymphatic malformation, PI3K/AKT/mTOR pathway, rapamycin, sirolimus

## Abstract

**Introduction:**

Kaposiform lymphangiomatosis (KLA) is an ultrarare disease characterized by abnormal lymphatic vessel proliferation due to hyperactivation of the Rat sarcoma virus (RAS) signaling pathway, resulting in multifocal lymphatic malformations (LMs) that can potentially involve multiple organ systems. Its distinctive histologic features include the presence of clusters of spindle cells expressing clusters of differentiation 31 (CD31), cluster of differentiation 34 (CD34), podoplanin (D2-40), prospero homeobox protein 1 (PROX1), and lymphatic vessel endothelial hyaluronan receptor 1 (LYVE1), along with abnormal lymphatic vessels. Sirolimus is recommended as a treatment option, particularly for patients without known mutations. This study aimed to examine the long-term therapeutic effects of sirolimus treatment in adult patients with KLA.

**Materials and methods:**

Between 2016 and 2024, KLA was diagnosed in three women, aged 21, 35, and 46 years, based on clinical presentation, laboratory findings, and imaging studies, and the diagnosis was confirmed by histological examination. The administration of sirolimus was initiated in all three cases at a therapeutic dose.

**Results:**

Treatment resulted in clinical improvement, partial regression, and subsequent stabilization of fluid volume in the pleural cavities, along with reductions in the severity of anemia and hypofibrinogenemia, accompanied by a decrease in D-dimer concentration. Sirolimus proved to be a relatively effective treatment in this group of KLA patients; however, some beneficial effects were not sustained after 5 years of follow-up. The most common side effects included mucositis and lipid disorders. We also reviewed the literature regarding sirolimus treatment in KLA patients.

**Conclusion:**

Overall, sirolimus in KLA patients is an effective treatment option with acceptable toxicity.

## 1 Introduction

Kaposiform lymphangiomatosis (KLA) is an ultra-rare congenital disease classified as a slow-flow complex lymphatic malformation (also known as a complex lymphatic anomaly [CLA]). The International Society for the Study of Vascular Anomalies (ISSVA) classifies anomalies into two main categories—developmental tumors and vascular malformations (1).

KLA is a disease entity at the borderline between vascular anomalies and tumor-like lesions. This disorder is most commonly observed in pediatric patients; therefore, experience with its management in the adult population remains limited. Reported cases in adults are extremely scarce in the medical literature, reflecting the exceptional rarity of the disease. It appears that both men and women are equally affected, although a predominance of male patients has been noted in some reports. The disorder usually manifests at birth or in early childhood. Dozens of cases have been described worldwide, the majority of which involve pediatric patients (2–12). Case reports of KLA in the adult population are presented in Table 1 (10–18). Overall, more than 70 confirmed KLA cases have been reported in the literature.

**Table 1:** Studies identifying the most common symptomatology associated with KLA in adult patients.

Authors	Sex	Age in years	Lesion location	Symptoms
Yoo et al., (38)	Male	35	Mediastinum, lungs, and pleura	Dyspnea, cough, and chest pain
Safi et al. (17)	Female	50	Mediastinum	Recurrent hemoptysis productive cough, chest pain, and mediastinal lymphadenopathy
Goyal et al., (39)	Female—2 Male—2	28 and 30 (F) both 19 (M)	Mediastinum and musculoskeletal system	Dyspnea, cough, and chest pain
Radzikowska et al. (15)	Female	33	Mediastinum, pleura, lung, pericardium, and lymph nodes	Chest pain, dyspnea, hemoptysis, coagulation disorders—elevated d-dimer concentration, and anemia
Radzikowska et al. (14)	Female	21	Mediastinum, pleura, lungs, and lymph nodes	Dry cough, dyspnea, fatigue, weight loss, and hemoptysis Coagulation disorders—elevated d-dimer concentration and decreased fibrinogen concentration
Foster et al. (36)	Female	18	Mediastinum, lungs, pleura, spleen, bones, and skin	Dyspnea and recurrent respiratory infections
Ospino and Brookmeyer (11)	Female	43	Mediastinum, pleura, pericardium, abdominal cavity, and gallbladder wall	Dyspnea, chest pain, chylothorax, and coagulation disorders—DIC
Nunes et al., 2023	Male	17	Cervical region, mediastinum, retroperitoneal space, and skeletal system	Fatigue, dyspnea, coagulation disorders—DIC, and pancytopenia

The pathophysiology of the disease is complex and not fully understood. Overactivation of the RAS gene-dependent signaling pathway, caused by somatic mutations—the most common of which is the p. Q61R variant—plays a key role in pathogenesis. This mutation induces over-phosphorylation via phosphoinositide kinase 3 (PI3K), which leads to the activation of protein kinase B (AKT). One of AKT’s functions is to activate the *mammalian target of rapamycin* (mTOR) kinase. The PI3K/AKT/mTOR pathway acts as a master regulator of many cellular processes, including cell metabolism, cell motility, angiogenesis, and cell proliferation. Enhanced mTOR signaling increases the expression of vascular endothelial growth factor (VEGF), a key regulator of angiogenesis and lymphangiogenesis. Disorders leading to hyperactivation of the PI3K/AKT/mTOR pathway result in excessive proliferation of mutation-laden cells (4, 5, 9, 12–18).

The clinical presentation is highly variable; the disease can take either a generalized or a localized form. Any system/organ can be involved; however, the most common locations of lesions are the mediastinum, lungs, bones, spleen, skin, and soft tissues. Due to the diverse organ involvement, the range of clinical manifestations is also broad. Patients with KLA most often present with hemoptysis, chylous effusions within body cavities, mediastinal lymphadenopathy, reduced exercise tolerance, dyspnea, or chest pain. Laboratory results may include abnormalities in the peripheral blood count, such as anemia and thrombocytopenia, elevated D-dimers, and reduced fibrinogen levels (14–18).

Microscopically, KLA is characterized by multifocal conglomerates of abnormal lymphatic vessels containing spindle cell clusters that express the CD31, CD34, D2-40, PROX1, and LYVE1 antigens. Pathomorphological examination is crucial for establishing the correct diagnosis (4, 5, 9, 12–18).

Numerous studies have indicated the effectiveness of sirolimus in the treatment of complex lymphatic anomalies, including KLA. The mechanism of action involves inhibition of the mTOR kinase, which is one of the key enzymes involved in the proliferation of cells of vascular origin (5, 19–29).

The purpose of this study was to examine the long-term efficacy of sirolimus treatment in adult KLA patients and to assess the correlation between serum D-dimer and fibrinogen levels and disease activity. In addition, a review of the literature regarding sirolimus treatment in KLA patients is provided.

## 2 Materials and methods

Between 2016 and 2024, KLA was diagnosed in three female patients in our department. Their median age was 33 years (range: 21 to 46 years). The diagnosis was based on clinical features, laboratory findings, and imaging and was supported by the histopathological assessment of surgical biopsy specimens taken from tumor masses in the mediastinum, lymph nodes, pleura, or lungs. Data on patients’ demographics, concomitant diseases, smoking habits, clinical symptoms, clinical signs, localization of lesions, pulmonary function test results, and cardiac echocardiography at the time of treatment initiation and during follow-up were collected.

Two cases were previously published as clinical images (14, 15).

For radiological assessment of lesions, magnetic resonance imaging (MRI) of the chest and the abdomen was preferred, although computed tomography and chest x-rays were also used when deemed appropriate by a radiologist. All radiological examinations were interpreted by an experienced radiologist (KB).

Microscopic analysis was performed by an experienced thoracic pathologist (MSz) using histopathological specimens obtained from lesions in the mediastinum, lymph nodes, lungs, or pleura. In one case, the microscopic image did not meet the criteria for KLA, and the final diagnosis was made based on clinical and radiological findings.

Pulmonary function tests were performed according to the joint guidelines of the American Thoracic Society and the European Respiratory Society. Lung volumes were measured via body plethysmography (MasterScreen software, ver. 4.65; Jeager, Wuerzburg, Germany), and the transfer factor of the lung for carbon monoxide (TL_CO_) was determined via the single-breath technique (30, 31).

Sirolimus was administered at a dose necessary to achieve a therapeutic drug concentration of 5–15 ng/mL. In addition, patients received bisphosphonates (sodium alendronate, once a month) and a low dose of oral steroids (5–10 mg/day). In patients who developed lipid disorders as an adverse reaction to sirolimus, statin therapy was initiated.

The effectiveness of the treatment was assessed based on radiological monitoring of the size of vascular lesions, clinical assessment, and laboratory parameters, including hemoglobin levels, platelet count, D-dimer serum concentration, and fibrinogen levels.

Adverse events were assessed using the Common Terminology Criteria for Adverse Events (CTCAE) version 5.0. Patients were assessed at the beginning of treatment, at 3, 6, and 12 months, and annually thereafter. Patients were also assessed whenever a significant problem developed.

The study adhered to all relevant tenets of the Declaration of Helsinki. All patients provided written informed consent for both treatment and publication.

## 3 Results

KLA was diagnosed in three female patients aged 21, 33, and 46 years. None of them were smokers, and none had any environmental exposure to pneumotoxic agents.

Patient No. 1 had a history of allergic sinusitis, colonization of *Streptococcus* spp. in the nasopharynx, and recurrent upper respiratory tract infections. Additionally, she had a history of pericarditis complicated by life-threatening cardiac tamponade during childhood. Laryngeal papillomatosis was diagnosed in patient No. 2, whereas patient No. 3 had undergone radioiodine treatment for toxic goiter and had been treated for many years for hypertension and colonic diverticulosis.

The time between the onset of first symptoms and diagnosis varied from 1 year to 8 years in patient No. 3, with a median duration of 2 years.

The most frequently reported symptoms were reduced exercise tolerance manifesting as exertional dyspnea (3/3), non-productive cough (2/3), recurrent hemoptysis (2/3), fatigue (3/3), subfebrile states (2/3), and peripheral edema (1/3; Table 2).

**Table 2:** Clinical symptoms in the studied group.

Patient characteristics	Patient No. 1	Patient No. 2	Patient No. 3
Age at diagnosis in years	21	33	46
Symptoms	Cough, hemoptysis, and progressive dyspnea weight loss	Low-grade fever, right-sided chest pain, and exercise intolerance	Cough, recurrent presence of pleural fluid, hemoptysis, dyspnea, and lower limb edema
Time from onset of symptoms to diagnosis	2 years	3 months	8 years
Duration of treatment	8 years with interruptions, including self-discontinuation of treatment between 2020 and 2022	9 years	5 years with multiple interruptions due to infections and exacerbation of acne-like lesions, edema,
Comorbidities	Allergic sinusitis history of pericarditis	Laryngeal papillomatosis connected to the pathogenic human papillomavirus (HPV) strain and cervical polyp resection with conization	Hyperthyroid goiter with post-radioiodine therapy, hypertension, and diverticulosis of the large intestine

In all cases, radiological examinations of the chest revealed lymphatic tumor masses in the mediastinum, with evidence of internal bleeding, along with multiple pulmonary and pleural lesions. In patient No. 1, pulmonary nodules, thickening of the interlobular septa, and foci of ground glass opacities were visualized. In patient No. 2, the right pleural cavity contained multiple collections of blood-like fluid, causing secondary atelectasis due to compression. Additionally, parenchymal thickening along the bronchovascular bundles was observed. In patient No. 3, bilateral pleural effusions with associated segmental pleural thickening, foci of atelectasis (most likely from compression), and thickening of the interlobular septa were revealed. Furthermore, enlargement of mediastinal and hilar lymph nodes was noted in all cases, along with previously described interlobular nodes in patient No. 2.

MRI examination in all patients revealed the presence of tumor-like masses in the mediastinum, consisting of conglomerates of abnormal lymphatic vessels. The dimensions of the lesions ranged from a few to several centimeters. In patient No. 1, a lesion measuring approximately 18 × 10 cm extensively occupied the entire mediastinum. In all cases, hemorrhagic foci with characteristic hyperintensity on T1-weighted images were identified, consistent with the presence of methemoglobin. Additionally, pleural fluid and small nodular lesions within the lung parenchyma were observed in all patients.

Splenic cystic lesions were also present in all patients described.

In patient No. 3, MRI also revealed lymphatic malformations in the abdominal cavity, appearing as a cluster of fluid-filled spaces located between the liver, the pancreas, and the spleen. The imaging findings indicated the presence of mesenteric lymphatic anomalies.

**Figure 1:**
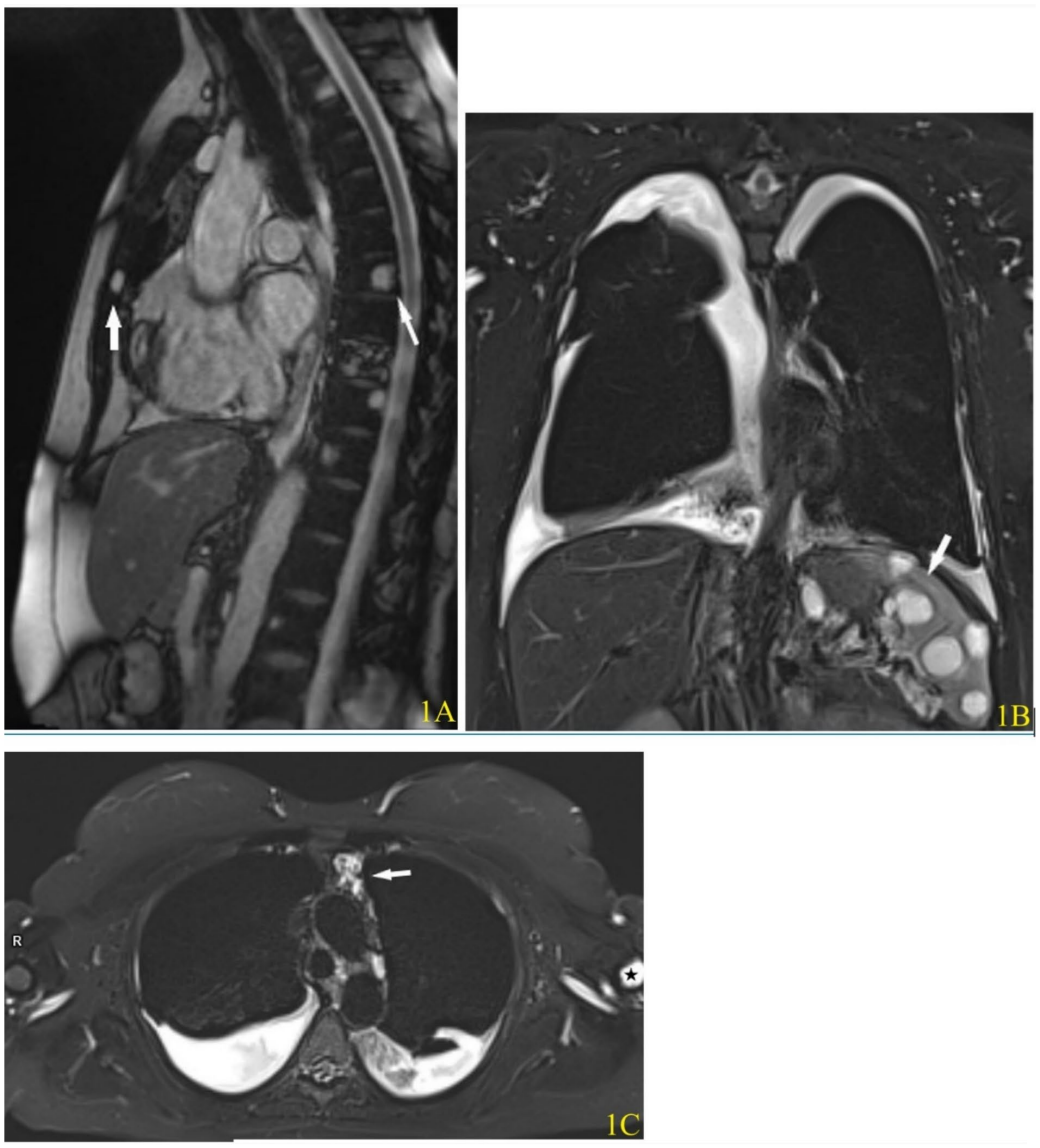
Magnetic resonance imaging of the chest of patient No. 3 at presentation. **(A)** T2-weighted TrueFISP image, sagittal view. Multiple lesions of T2 hyperintensity are present in the vertebral bodies and sternum (arrow). **(B)** T2-weighted short tau inversion recovery (STIR) image, coronal view. Multiple cystic lesions with high T2 signal are visible in the spleen (arrow). **(C)** T2-weighted STIR image, axial view. Bilateral pleural effusion and a bright-signal lymphatic malformation are present in the mediastinum (arrow). A hyperintense bone lesion is also present in the left humerus is also present (asterisk).

**Figure 2:**
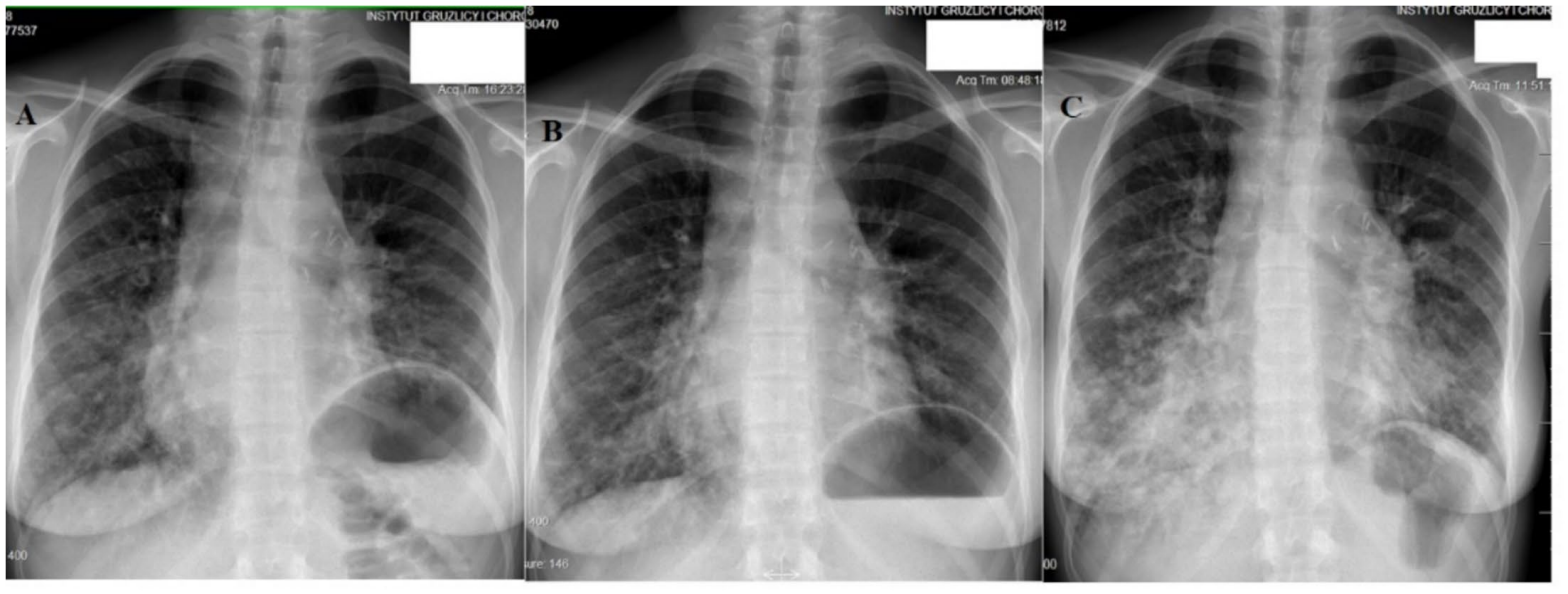
Patient No. 1’s chest X-ray examinations. **(A)** Posteroanterior (PA) chest X-ray image at the time of treatment initiation shows bilateral opacities, mostly in the middle and lower fields of the right lung, and smaller quantities in the left lung. The outline of the mediastinum and pulmonary hila is widened, and the left diaphragm dome is elevated. **(B)** PA chest X-ray image after 1 year of sirolimus treatment shows a distinct reduction in bilateral lung opacities. The outline of the mediastinum and pulmonary hila is widened. The left diaphragm dome is elevated. **(C)** PA chest X-ray image after 5 years of sirolimus treatment (with significant treatment-free periods due to non-adherence) shows massive, bilateral progression of lung opacities, predominantly in the lower and middle fields of the right lung.

**Figure 3:**
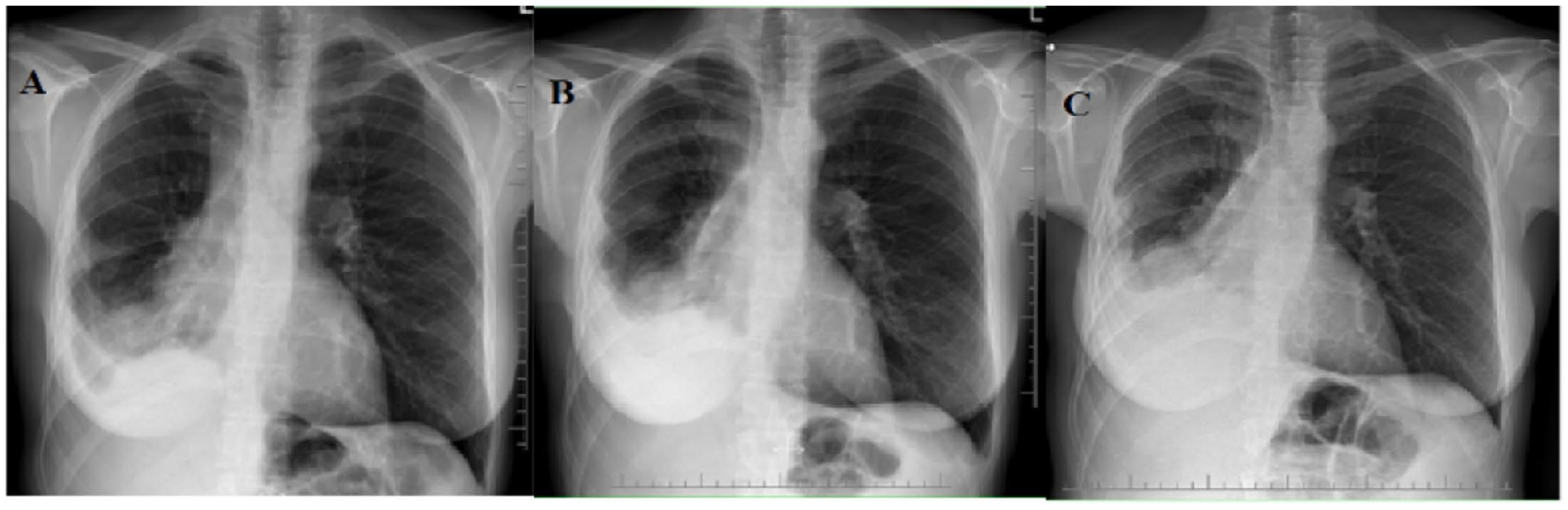
Patient No. 2’s chest X-ray examinations. **(A)** PA chest X-ray image at the time of treatment initiation shows a collection of fluid in the right pleural cavity; irregular interstitial thickening in the lower right lung is also visible. **(B)** PA chest X-ray image after 1 year of sirolimus treatment shows stabilization of the fluid collection in the right pleural cavity. **(C)** PA chest X-ray image after 5 years of sirolimus treatment shows a slight increase in the amount of fluid in the right pleural cavity.

**Figure 4:**
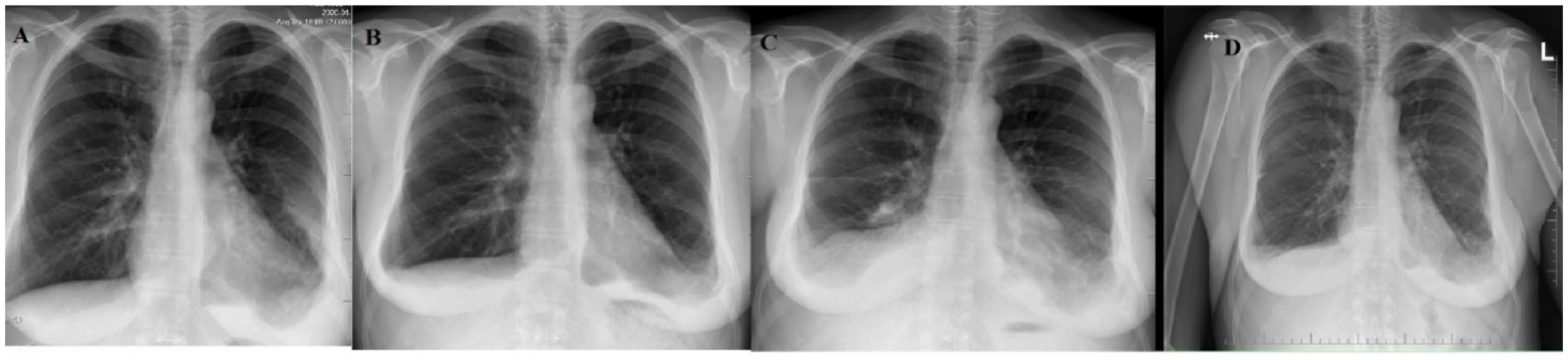
Patient No. 3’s chest X-ray examinations. **(A)** PA chest X-ray image at the time of treatment initiation shows single opacities in the lower field of the right lung with a small amount of fluid in the right pleural cavity and fluid in the left pleural cavity. **(B)** PA chest X-ray image after 1 year of sirolimus treatment shows partial regression of fluid in the left pleural cavity. **(C)** PA chest X-ray image after 2 years of sirolimus treatment shows an increased amount of fluid in the left pleural cavity, reappearance of fluid in the right pleural cavity, and increased consolidation in the lower field of the right lung. **(D)** PA chest X-ray image after 5 years of sirolimus treatment shows partial regression of fluid in the right pleural cavity and stabilization of fluid in the left pleural cavity.

In patients Nos. 1 and 3, magnetic resonance imaging detected bone marrow lesions. In patient No. 1, these were identified in the vertebral bodies, sacrum, iliac bone, and femur, whereas in patient No. 3, lesions were observed in the thoracic vertebral bodies, sternum, left humeral shaft, and ribs (Table 3). The radiological images are shown in Figures 1–4.

**Table 3:** Organ involvement and laboratory findings in the presented group of KLA patients.

Clinical and laboratory features	Patient No. 1	Patient No. 2	Patient No. 3
Involved organs			
Lungs	+	+	+
Pleura	+	+	+
Mediastinum	+	+	+
Spleen	+	+	+
Other	Liver	Lymph nodes (mediastinal, hilar, and interlobular)	Lymphatic malformations of the mesentery and aortic arch region and
	Lateral neck cyst, and Bones		Bones
D-dimer (ng/mL), initial assessment; normal <500	14,905	5,478	2,650
Fibrinogen (g/l), initial assessment; normal range: 2.2–4.9	1,61	2,37	1,98

Ventilatory parameters were mildly reduced in all patients (Table 4). Transfer factor of the lung for carbon monoxide (TL_CO_) was slightly decreased in patients No. 1 and 3, while patient No. 2 had a moderately reduced TL_CO_.

**Table 4:** Ventilatory parameters at baseline and during treatment in the studied group of KLA patients.

Parameter	Patient No. 1	Patient No. 2	Patient No. 3
Forced expiratory volume in 1 s (FEV₁) [L/s, % predicted]			
Baseline	2.17 (67%)	2.06 (66%)	1.83 (61%)
After 1 year	1.86 (58%)	2.15 (69%)	1.93 (65%)
After 2 years	2.09 (65%)	2.33 (77%)	1.84 (63%)
After 5 years	1.39 (48%)	1.87 (64%)	1.68 (58%)
VC [L; % predicted]			
Baseline	2.51 (69%)	2.6 (70%)	2.55 (69%)
After 1 year	2.42 (66%)	2.55 (69%)	2.57 (70%)
After 2 years	2.74 (75%)	2.84 (79%)	2.52 (69%)
After 5 years	2.02 (56%)	2.45 (76%)	2.48 (68%)
TLC [L; % predicted]			
Baseline	3.88 (81%)	4.05 (83%)	3.93 (76%)
After 1 year	3.72 (78%)	3.86 (79%)	5.17 (95%)
After 2 years	4.21 (88%)	3.93 (81%)	4.42 (86%)
After 5 years	-	4.81 (99%)	-
TL_CO_ [mL/min/mmHg; % predicted]			
Baseline	5.74 (63%)	5.02 (57%)	6.19 (72%)
After 1 year	5.56 (61%)	4.6 (52%)	5.91 (69%)
After 2 years	5.85 (64%)	5.34 (62%)	5.72 (67%)
After 5 years	-	-	-

Due to COVID-19 pandemic restrictions, body plethysmography could not be performed at certain checkpoints.

**Figure 5:**
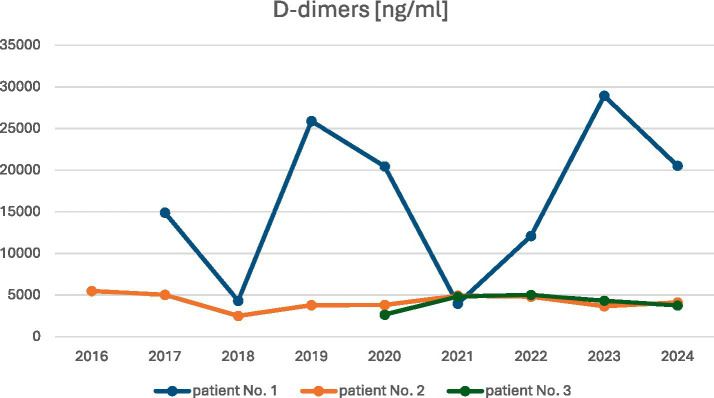
Patients’ D-dimer levels during observation.

**Figure 6:**
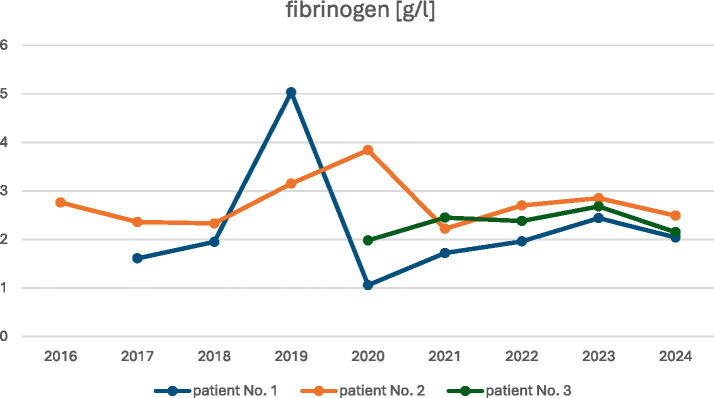
Patients’ fibrinogen concentration during observation.

Elevated D-dimer levels were observed in all patients, with a median concentration of 7,678 ng/mL (range: 2650–14,905 ng/mL) and a normal range of <500 ng/mL (Figure 5). Serum fibrinogen concentration levels were reduced in two patients—No. 1 and No. 3, with a median level of 1.8 g/L (range: 1.61–1.98 g/L); the normal range was 2.2–4.9 g/L (Figure 6).

**Figure 7:**
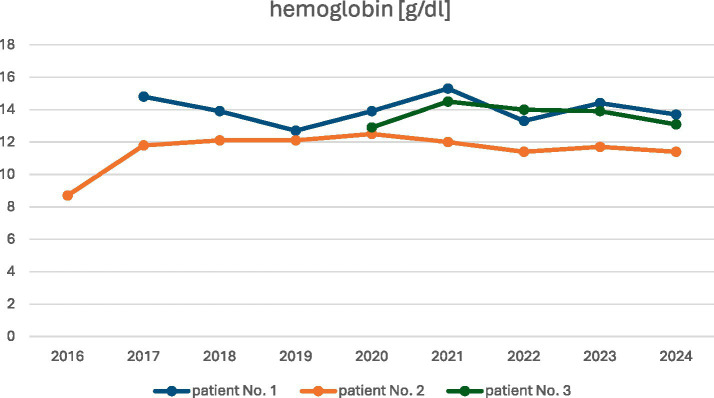
Patients’ hemoglobin concentration during observation.

At the time of diagnosis, moderate anemia with a hemoglobin level of 8.7 g/dL was found in patient No. 2. In the remaining patients, hemoglobin levels were within or at the lower limit of the normal range (Table 2; normal range: 11.2–15.7 g/dL; Figure 7).

Patient No. 1 was observed for more than 8 years; however, she took sirolimus with multiple long interruptions, which likely resulted in its incomplete efficacy.

The other patients were treated for 9 years (patient No. 2) and 5 years (patient No. 3). Patient No. 2 also periodically discontinued treatment or received reduced doses due to recurrent upper respiratory tract infections; however, the interruptions were brief. Patient No. 3 also had experienced some interruptions in treatment due to mild upper respiratory tract infections, edema of subcutaneous tissue, and acne-like lesions.

After 1 year of treatment, two patients experienced a reduction in mediastinal lesion dimensions (patients Nos. 2 and 3); however, only in patient No. 2 did the reduction meet the criteria for a partial response (PR) to treatment. In patient No. 1, lymphatic malformation masses progressed, most likely due to treatment interruptions. This patient also experienced a significant increase in D-dimers and mild anemia. In patient No. 2, D-dimer levels decreased with an accompanying increase in fibrinogen levels. In patient No. 3, after 1 year of treatment, an increase in D-dimer levels to 25,886 ng/mL was observed, without an accompanying decrease in fibrinogen levels, while a concomitant elevation in inflammatory markers was noted—(C-reactive protein [CRP]: 227.7 mg/L [normal range <5 mg/L] and procalcitonin: 0.24 ng/mL [normal range <0.5 ng/mL]). At this time, the patient was diagnosed with sepsis caused by *Staphylococcus aureus* MSSA and *Staphylococcus epidermidis* originating from the pharyngeal lymphatic ring. In subsequent follow-ups, D-dimer levels decreased, along with a decrease in CRP serum concentration. Two years of therapy resulted in stabilization of laboratory parameters in two patients (Nos. 2 and 3). Patient No. 1 continued to have significantly elevated D-dimers with decreased fibrinogen levels. Stabilization of mediastinal lesions persisted in patients Nos. 2 and 3, while a slight regression of pulmonary lesions was observed in patient No. 1.

After a 5-year follow-up, two patients (Nos. 1 and 3) demonstrated progression of pulmonary lesions, characterized by increased fluid accumulation in the pleural cavities and an increase in nodular lesions. However, patient No. 2 showed stabilization of mediastinal lesions. Patient Nos. 2 and 3 continued to exhibit stable laboratory parameters, namely hemoglobin, D-dimers, fibrinogen, and platelets. In patient No. 1, an increase in D-dimer concentration was observed, with fibrinogen levels at the lower limit of normal (although the serum fibrinogen concentration was higher than that at previous control points), while a decreasing trend in D-dimer concentration had been observed at previous checkpoints.

The observed changes in the values of laboratory parameters, such as D-dimers, fibrinogen, and hemoglobin levels, over the years are shown in Figures 5–7.

## 4 Adverse events

The most common adverse events of sirolimus treatment were lipid disorders, experienced by all patients (none more severe than grade 2 and manageable). There was also an increased susceptibility to infections, with patient Nos 1 and 3 experiencing grade 3 or 4 infections. Moreover, all patients experienced between 2 and 4 mild upper respiratory tract infections per year during treatment. Skin and mucosal lesions were observed in patient No. 3 (1/3), requiring a periodic reduction in the sirolimus dose. In patient No. 3, the peripheral edema observed before treatment was exacerbated following initiation of sirolimus but was managed with a slight reduction in sirolimus dosage and physiotherapy. In patient No.1, mild lymphopenia was observed during treatment. All adverse events are summarized in Table 5.

**Table 5:** Adverse events in KLA patients.

Adverse event	Patient No. 1	Patient No. 2	Patient No. 3
1st–2nd grade infections	**+** 16 episodes of URTI	**+** Pneumonia, laryngeal papillomas, and 5 episodes of URTI	**+** 12 episodes of URTI
3rd–4th grade infections	**+** MSSA sepsis after 2 years of treatment	**−**	**+** COVID-19 and pertussis
Hypercholesterolemia of at least 2nd grade	**−**	**+**	**−**
Hypertriglyceridemia of at least 2nd grade	**−**	**+**	**−**
Skin and/or mucosal changes of at least 2nd grade	**−**	**−**	**+**
Edema—2nd grade	**−**	**−**	**+**
Lymphopenia 1st—2nd grade	**+** 3 episodes	**−**	**−**

1. URTI, upper respiratory tract infection. 2. MSSA, meticillin-sensitive *Staphylococcus aureus*. 3. All patients experienced grade 1 hypercholesterolemia. 4. All patients experienced grade 1 hypertriglyceridemia.

## 5 Discussion

**Figure 8:**
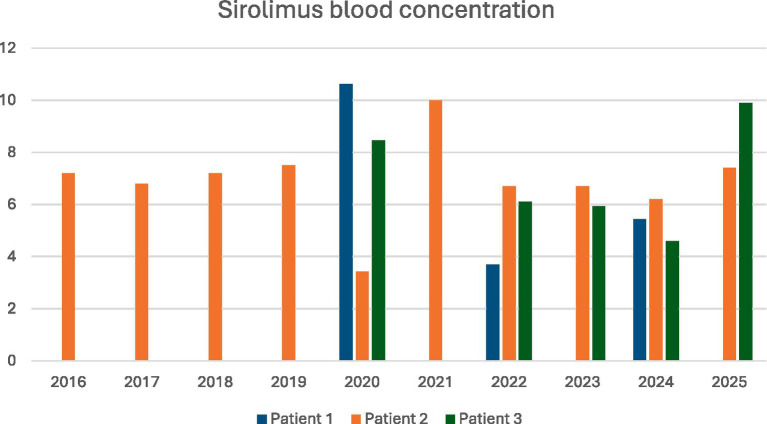
Patients’ mean sirolimus blood concentrations. Sirolimus doses: Patient No. 1–2 mg. Patient No. 2–3 mg. Patient No. 3–2 mg until 2022, then escalated to 2 mg on even days and 3 mg on odd days.

Kaposiform lymphangiomatosis is extremely rare in the adult population, and the number of reports on its treatment and management remains limited (Figure 8).

In this study, we present, to our knowledge, for the first time a group of adult KLA patients with such long-term observations of sirolimus treatment outcomes. The efficacy of sirolimus treatment was satisfactory, and the observed side effects were acceptable. Treatment failure and progression of mediastinal lymphatic malformation size observed in patient No. 1 were most likely associated with non-adherence to medical recommendations and the patient’s arbitrary discontinuation of the drug. Consistent with the previously cited articles (5, 19–28), we also observed beneficial effects of sirolimus initiation in our patients, such as stabilization or regression of vascular mass size following treatment initiation. Notably, there was a clear correlation between the reduction of hemangioma masses and the secondary stabilization of coagulation parameters (D-dimers and fibrinogen), along with normalization of hemoglobin levels and a decreased bleeding tendency (patients Nos. 2 and 3, in the early phase of treatment, also patient No. 1). In addition, lung ventilation parameters improved in patient No. 3. Among all patients, previously reported dyspnea and chest pain decreased or resolved. In patient No. 3, who was initially diagnosed with peripheral edema, a significant reduction was noted. In all patients, a significant reduction in disease symptoms, such as dyspnea, chest pain, and fatigue, was observed. Furthermore, the fact that patient No. 1 experienced periodic progression of lymphatic malformation size, re-exacerbation of coagulation disorders, and worsening of general condition after drug discontinuation further supports the beneficial effect of sirolimus.

KLA is a disease primarily diagnosed in the pediatric population, and data concerning adults are scarce. It is a systemic, multifocal, and multiorgan disease with a frequently aggressive course and poor prognosis. The involvement of multiple potential organs presents a broad range of clinical symptoms, which are often non-specific and may pose diagnostic difficulties (2–18).

In individual studies describing KLA in adults, the disease primarily involved the mediastinum (including the lungs, pleura, pericardium, and lymph nodes). The main complaints reported by the patients included dyspnea, initially on exertion, chest pain, cough, or recurrent hemoptysis. Coagulation disorders, associated with excessive clotting within pathological lymphatic vessels, are also common. The clinical, radiological, and laboratory manifestations of the presented patients did not differ from other cases presented in the literature, although we did not observe thrombocytopenia, coagulation disorders, or intravascular coagulation syndrome, as described in other studies (2–18). The aforementioned disorders occur as a result of increased bleeding from pathological vascular masses and, consequently, excessive coagulation, during which clotting factors are consumed.

The severity of the disorders correlated with the response to sirolimus treatment, and these parameters may serve as sensitive markers of disease activity.

The first description of this clinical entity was made by Croteau et al. (6) based on a retrospective series of 20 patients with distinctive histopathology. Croteau et al. found that the median age of onset was 6.5 years (range: 0–44 years), with 5-year survival of 51% and overall survival of 34%, and a mean interval from diagnosis to death of 2.75 years (range: 1–6.5 years). It was also the first study to analyze different treatment modalities in this group of patients. Among them, five patients were treated with sirolimus, with treatment durations ranging from 5 days to 18 months. Four of them had sustained clinical improvement after 6 months, with three maintaining this improvement for more than a year.

Sirolimus is a medication that has been used in the treatment of different vascular malformations for years; however, the number of studies specifically addressing KLA remains limited (5, 11, 19–28), and these are presented in Table 6.

**Table 6:** Studies reporting the outcomes of sirolimus treatment in patients with KLA.

Authors	Patient number and sex	Approx. treatment duration (months)	Treatment outcomes	Remarks
Croteau et al. (6)	5 patients with KLA treated with sirolimus	6	Improvement of symptoms	-
Adams et al. (5)	7 patients with KLA overall, 60 patients with CLA: 35 F, 25 M 10 adults (17%), 50 children (83%)^1^	6 11	6 PR and 1 PD After 6 courses: 47 PR, 3 SD, and 7 PD; after 12 courses: 45 PR and 8 PD	7 patients did not complete the full treatment 2 discontinued due to drug-related toxicity
Zhou et al. (20)	7 patients: 6 M, 1 F	6–42	3 PR, 3 SD, and 1 PD	One patient with PD died
Ji et al. (29)	4 patients	12 24	2 PR, 1 SD, and 1 PD	-
Nunes et al. (2023)	1 M, 17 years	48	Stabilization of lymphatic malformation size and laboratory parameters; improved quality of life	No significant adverse events were observed
Ospino and Brookmeyer (11)	1 F, 43 years	12	Significant clinical and radiological improvement	No significant adverse events were observed
Bilny-Paluch et al. (presented data)	3 F, 21–46 years	60–108	Significant clinical and radiological improvement; PD in one patient due to treatment withdrawal	One episode of severe infection, likely associated with prior carriage of *Streptococcus pyogenes*

1. F, female; M, male. 2. PR, partial response. 3. SD, stable disease. 4. PD, progressive disease. 5. GLA, generalized lymphatic anomaly. 6, KLA, kaposiform lymphangiomatosis. ^1^This is ordinal numeral.

Adams et al. described the effect of 1-year sirolimus treatment in patients with complex vascular anomalies in a group of 61 patients (5). The group included patients in different age ranges: 55% of patients were children below the age of 9 years, 28% were adolescents between 10 and 19 years, and 17% were adults aged 20–29 years. The enrolled diagnoses (according to the ISSVA 1997 classification) included phosphatase and tensin homolog deleted on chromosome 10 (PTEN-associated vascular anomaly) in 6 patients, generalized lymphatic anomaly (GLA) in 7 patients, Gorham–Stout disease in 3 patients, kaposiform lymphangiomatosis in 7 patients, microcystic lymphatic malformation in 5 patients, kaposiform hemangioendothelioma in 13 patients, lymphangiectasia in 3 patients, and venous lymphatic malformation in 3 patients. Fifty-seven patients were evaluated after 6 courses of treatment, and 53 completed 12 courses. Among these, 47 patients achieved a partial response (PR, 87%), 3 had stabilization of stable disease (SD, 5%), and 8 had progressive disease (PD, 12%). In a subgroup of patients with KLA, 6 months of treatment resulted in partial response in 5 cases (71%), stable disease in 1 case (14%), and progressive disease in 1 case (14%). After 12 months, 6 patients achieved PR (86%) and 1 had PD (14%). In addition to radiological response, changes in quality of life and functional impairment were also evaluated. After 1 year in the group of 53 patients, 39% had normalization of quality of life, 52% had an improvement in self/proxy-reported pediatric quality-of-life inventory scale, 16% had stable disease, and 1 patient had progressive disease. The functional impairment score improved in 80% of the patients, while the remaining patients showed stabilization. The most common side effects included bone marrow toxicity (50%), infections (37%), dyslipidemia (hypercholesterolemia and hypertriglyceridemia), hyperglycemia (18%), and gastrointestinal symptoms (17%). The authors highlighted the fact that the most prominent effects were observed among younger patients, suggesting a benefit of early treatment initiation.

In a study by Zhou et al., the effect of sirolimus treatment was analyzed in a group of seven patients with KLA (aged 4 months to 6 years) treated prospectively (20). Three of them achieved partial response, three had stable disease, and one had progressive disease (PD). The authors also pooled the data from 17 previously described cases from the literature, with their group assessing the response in a group of 24 patients. Overall, 58.3% of patients had a partial response, 25% had stable disease, and 16.7% experienced progressive disease.

The outcomes of sirolimus treatment on different vascular anomalies were analyzed in a multicenter, prospective study by Ji et al. The group consisted of patients with different CLAs, simple lymphatic anomalies, venous anomalies, and combined anomalies. The primary outcome was the change in anomaly volume on MRI. There were four patients with KLA: two achieved PR, 1 had SD, and 1 experienced progressive disease after 12 months of treatment. After 24 months, the results remained the same. The secondary outcomes included the severity of the disease after 12 months, which improved in three patients with KLA, and quality of life, which improved in two patients.

Ozeki et al. presented the effect of 6 months of sirolimus treatment in a group of 20 patients with CLA analyzed prospectively (21). The study included three patients with KLA: one adult (20 years old) who had a partial response, and two children, with one having PR and the other one having SD. Overall, 10 patients had a partial response. In addition, the quality of life improved. Another 10 patients had SD with no significant benefits in terms of quality of life or disease severity after sirolimus treatment. Eighty percent of patients experienced adverse events such as stomatitis, infections, and hyperlipidemia; however, these adverse events did not result in treatment discontinuation.

The efficacy of sirolimus treatment has also been reported in several case reports. Ospino et al. published a case of a 43-year-old woman with recurrent chylothorax, chylous pericardial and peritoneal effusions, and chronic disseminated intravascular coagulation (DIC), requiring frequent hospitalizations and blood transfusions (11). After 12 months of sirolimus treatment, she had a radiological response, resolution of DIC, and improvement in pulmonary function tests. No adverse events were reported. Another case report by Pereira-Nunes et al. described a 17-year-old patient with giant lymphatic malformation presenting with coagulopathy, requiring frequent blood transfusions due to severe anemia, which subsided after 4 years of sirolimus treatment, along with stabilization of the malformation size (16).

Moreover, sirolimus has been reported as a successful treatment option in several studies that included various CLAs other than KLA (22, 29, 32–34). As these diseases share similar pathophysiology, overlapping clinical features, and responsiveness to drugs targeting the PI3K/AKT/mTOR pathway, this further supports the use of sirolimus in KLA patients.

Recently, promising reports of using other drugs targeting specific mutations in the treatment of complex lymphatic anomalies have been published. Canaud et al. presented a group of 57 patients with PIK3CA-related overgrowth spectrum (PROS) treated with alpelisib (35). Among 32 patients with complete follow-up for endpoint analysis, 60% had a radiological response, and 90.9% showed improvement in symptoms, with mild side effects (hyperglycemia in 12.3% and mucosal ulcerations in 10.5% of patients). Promising reports have also been published concerning the use of the mitogen-activated protein kinase kinase (MEK) inhibitor—trametinib—in patients with mutations in the RAS pathway (36, 37). Foster et al. presented a case of an 18-year-old patient suffering from KLA since the age of 6 years, who was initially treated with vincristine and interferon and then with sirolimus for 8 years. At the age of 18 years, the patient decided to discontinue rapamycin therapy, which shortly after led to deterioration of her overall condition. Despite resuming therapy with sirolimus and prednisone, her pulmonary functional parameters steadily deteriorated. The NRAS p. Q61R mutation was identified, and she was subsequently successfully treated with trametinib (36).

## 6 Study limitations

The limitations of this study include its retrospective design, small sample size, non-compliance with treatment in the case of patient No. 1, and limited data due to COVID-19 restrictions, which reduced the number of follow-up visits. Moreover, no genetic tests for known molecular variants associated with KLA were performed, which would have been valuable for patient profiling and assessing potential connections to treatment response. However, the rarity of the disease should be considered when interpreting the results.

Further research is needed to validate these findings.

## 7 Conclusion

Sirolimus treatment appears to be an effective and acceptable option for managing KLA, although further research and development of more effective therapies are needed.

## Data Availability

The original contributions presented in the study are included in the article/supplementary material; further inquiries can be directed to the corresponding author.

## Glossary

AKT: protein kinase B
CD31: cluster of differentiation 31
CD34: cluster of differentiation 34
COVID-19: coronavirus disease-2019
CRP: C- reactive protein
CTC AE: Common Terminology Criteria for Adverse Events
CVM: congenital vascular malformations
D2-40: podoplanin
DIC: disseminated intravascular coagulation
CLA: complex lymphatic anomalies
GLA: generalized lymphatic anomaly
ISSVA: International Society for the Study of Vascular Anomalies
KLA: kaposiform lymphangiomatosis
KTS: Klippel–Trenaunay syndrome
LM: lymphatic malformation
LYVE1: lymphatic vessel endothelial hyaluronan receptor 1
MEK: also known as MAP2K- mitogen-activated protein kinase kinase
MRI: magnetic resonance imaging
MTOR: mammalian target of rapamycin kinase
PA: posteroanterior
PD: progressive disease
PedsQL: Pediatric Quality of Life Inventory
PI3K: phosphoinositide kinase 3
PR: partial response
PROS: PIK3CA-related overgrowth spectrum
PROX1: prospero homeobox protein 1
PTEN: phosphatase and tensin homolog deleted on chromosome 10
PTHS: PTEN hamartoma tumor syndrome
SD: stable disease
TL_CO_: transfer factor of the lung for carbon monoxide
VEGF: vascular endothelial growth factor
